# Subependymal Zone-Derived Oligodendroblasts Respond to Focal Demyelination but Fail to Generate Myelin in Young and Aged Mice

**DOI:** 10.1016/j.stemcr.2017.01.007

**Published:** 2017-02-09

**Authors:** Ilias Kazanis, Kimberley A. Evans, Evangelia Andreopoulou, Christina Dimitriou, Christos Koutsakis, Ragnhildur Thora Karadottir, Robin J.M. Franklin

**Affiliations:** 1 Wellcome Trust-MRC Cambridge Stem Cell Biology Institute, University of Cambridge, Cambridge CB2 0AH, UK; 2 Lab of Developmental Biology, Department of Biology, University of Patras, Patras 26500, Greece

**Keywords:** oligodendrocyte progenitor cell, oligodendroblast, OPC, subependymal zone, subventricular zone, myelin, myelination, corpus callosum, ageing, neural stem cell, demyelination, remyelination

## Abstract

Two populations of oligodendrogenic progenitors co-exist within the corpus callosum (CC) of the adult mouse. Local, parenchymal oligodendrocyte progenitor cells (pOPCs) and progenitors generated in the subependymal zone (SEZ) cytogenic niche. pOPCs are committed perinatally and retain their numbers through self-renewing divisions, while SEZ-derived cells are relatively “young,” being constantly born from neural stem cells. We compared the behavior of these populations, labeling SEZ-derived cells using hGFAP:Cre^Ert2^ mice, within the homeostatic and regenerating CC of the young-adult and aging brain. We found that SEZ-derived oligodendroglial progenitors have limited self-renewing potential and are therefore not bona fide OPCs but rather “oligodendroblasts” more similar to the neuroblasts of the neurogenic output of the SEZ. In the aged CC their mitotic activity is much reduced, although they still act as a “fast-response element” to focal demyelination. In contrast to pOPCs, they fail to generate mature myelinating oligodendrocytes at all ages studied.

## Highlights

• SEZ-derived cells in the CC are oligodendroblasts and not OPCs
• Oligodendroblasts have limited self-renewal capacity and do not make myelin
• Oligodendroblasts respond rapidly after demyelination
• Aging does not affect the oligodendroblast-pOPC balance

## Graphical Abstract

**Figure:**
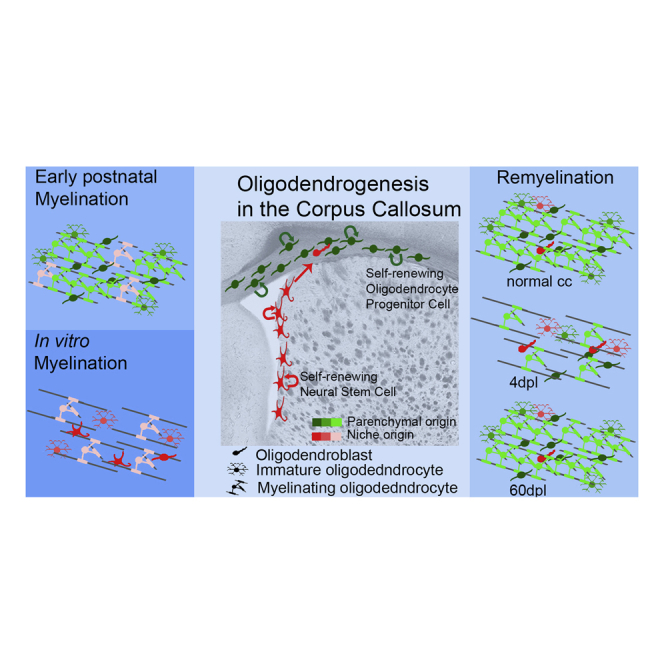

## Teaser

Franklin, Kazanis, and colleagues compare the two oligodendrogenic pathways that co-exist in the corpus callosum. They demonstrate that, irrespective of age, adult neural stem cells generate oligodendroblasts; i.e., progenitors with limited self-renewal capacity that respond rapidly to focal demyelination but eventually fail to generate new myelin, which are different to parenchymal oligodendrocyte progenitor cells that drive remyelination.

## Introduction

Myelination of the CNS of mice occurs mainly within the first 4 post-natal weeks when oligodendrocyte progenitor cells (OPCs) differentiate into myelin-forming oligodendrocytes. After this period, adult OPCs of divergent developmental origins remain scattered throughout the CNS parenchyma (Crawford et al., 2016). Their density is regulated via cell-contact inhibition and controlled proliferation (Hughes et al., 2013, Sim et al., 2002), their properties are origin- and region-dependent (Crawford et al., 2016, Dimou et al., 2008, Power et al., 2002, Tripathi et al., 2011, Young et al., 2013) and their cell cycle slows with aging (Young et al., 2013). An anatomically confined source of oligodendroglial lineage cells in the adult brain is the cytogenic niche located in the subependymal zone (SEZ, also called the ventricular-subventricular zone) of the lateral walls of the lateral ventricles (Jablonska et al., 2010, Menn et al., 2006, Nait-Oumesmar et al., 2007, Ortega et al., 2013). Neural stem cells (NSCs) residing therein divide infrequently to primarily generate neurons via transit-amplifying progenitors. Based on evidence produced from different experimental models of demyelination (Capilla-Gonzalez et al., 2014, Nait-Oumesmar et al., 2008, Xing et al., 2014), SEZ-driven oligodendrogenesis is considered as a potential alternative source of OPCs for the treatment of chronic demyelinating disorders, such as multiple sclerosis, although this capacity has not always been confirmed (Guglielmetti et al., 2014). What remains unclear is whether SEZ-derived progenitors mix with and replenish (or enlarge) the general OPC population, or if they generate myelin specifically in response to demyelination (Agathou et al., 2013). SEZ-derived and parenchymal OPCs (sezOPCs and pOPCs, respectively) differ not only in their cellular origin but also in their cellular age, since pOPCs are committed during the early post-natal period and retain their numbers through self-renewing divisions, while new sezOPCs are constantly born within the microenvironment of the niche. Based on previous work suggesting that adult pOPCs behave differently to perinatal OPCs (Windrem et al., 2004, Wolswijk and Noble, 1989), and that a fraction of aging pOPCs expresses markers of senescence (Kujuro et al., 2010), we compared the behavior of sezOPCs and pOPCs, in both homeostatic and regenerating tissue in the young and aged brain.

## Results

### sezOPCs are Labeled in hGFAP:Cre^ERT2^ x Rosa26:EYFP Mice

**Figure 1:**
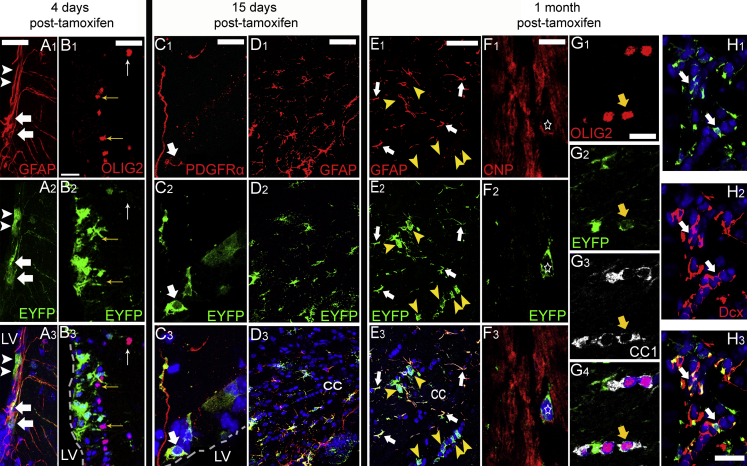
Expression of Oligoendroglial Markers in EYFP+ Cells of the SEZ and CC (A–H) At 4 days post tamoxifen, expression of EYFP was induced within the SEZ, in GFAP+ astrocytes, and in scattered ependymal cells, arrows and arrowheads, respectively, in (A), but no co-expression with Olig2 was detected (B): yellow arrows indicate OLIG2+ cells in the SEZ, white arrow an OLIG2+ cell in the striatum. At 15 days, double EYFP+/PDGFRα+ cells were detected in the SEZ (C), white arrow. The majority of EYFP+ cells in the CC co-expressed GFAP (D). At 1 month, in the CC EYFP+/GFAP− cells were detected, yellow arrowheads in (E), near EYFP+/GFAP+ astrocytes (white arrows). EYFP+ oligodendrocytes are indicated by the star in (F) (CNPase+) and the arrow in (G) (CC1+). At the same time point EYFP+/DCX+ neuroblasts were present in the SEZ, arrows in (H). Scale bars, 50 μm (A, B, D, and E); 20 μm (C, F, G, and H). The dashed line in (B) and (C) indicates the wall of the lateral ventricle (LV).

**Figure 2:**
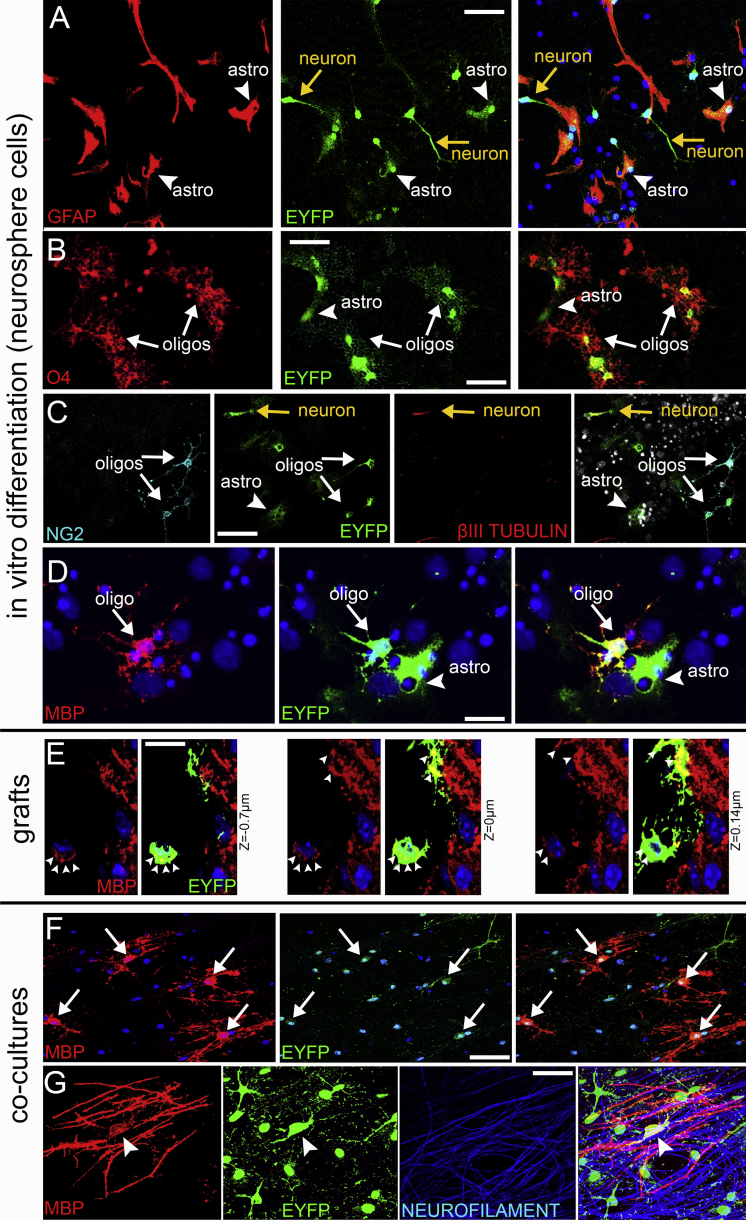
Differentiation Potential of Niche-Derived Cells (A) EYFP+/GFAP+ astrocytes and EYFP+ neurons (judged by morphology). (B) EYFP+/O4+ oligodendrocytes near an EYFP+ astrocyte (judged by morphology). (C) EYFP+/NG2+ OPCs are shown near an EYFP+/βIII-tubulin+ neuron and EYFP+ astrocytes. (D) An MBP+/EYFP+ cell is shown next to two EYFP+ astrocytes. (E) After grafting SEZ-derived neurosphere cells in 21 day old mice, EYFP+/MBP+ cells were detected in the CC (three different optical sections of an EYFP+ cell are shown and MBP+ areas of the cell body and of the processes are indicated by white arrowheads). (F) Co-cultures of neurosphere cells and dorsal root ganglion cells: myelinating oligodendrocytes are generated (white arrows) with strong expression of EYFP in cell bodies. (G) An EYFP+ myelinating oligodendrocyte is shown in high magnification (the arrowhead indicates the cell body). Note the strong expression of EYFP in the cell body and in some processes and the alignment of MBP+ processes with neurofilament+ axons. Scale bars, 30 μm (A–C, and F); 20 μm (D, E); 10 μm (G).

Adult NSCs express glial acidic fibrillary protein (GFAP) (Doetsch et al., 1999), the expression of which decreases abruptly in downstream progenitors, such as neuroblasts (Pastrana et al., 2009). Here, we used hGFAP:Cre^ERT2^ x ROSA26:EYFP double transgenic mice (Hirrlinger et al., 2006) (details on validation of alternative labeling strategies and of labeling efficiency are provided in the Supplemental Experimental Procedures). Administration of tamoxifen, assessed initially at 4 days post administration (n = 4), induced recombination in GFAP+ astrocytes throughout the adult gray and white matter (not shown) and in sporadic (<10%) of ependymal cells. In the SEZ and the corpus callosum (CC) the majority of EYFP+ cells co-expressed GFAP with no co-expression of oligodendroglial lineage markers and only limited expression of the neuroblast marker DCX (Figures 1A, 1B, S1E, and S1F; Table S1). At 15 days post tamoxifen (n = 4) cells expressing PDGFRα and OLIG2 were found in the SEZ (Figure 1C), and EYFP+OLIG2+ or EYFP+SOX10+ cells were also observed in the CC (Figures 1D and 4D; Movie S1). At 30 days (n = 6), the distribution of DCX+ neuroblasts and OLIG2+ cells within the SEZ pool of EYFP+ cells reflected the distribution of these cells in the whole SEZ (Figures 1H and S1G–S1I; Table S1), and significantly more EYFP+/GFAP− cells appeared within the supraventricular fragment of the CC (Figures 1E, 4D, S2C, and S2D; Table S1). These cells also co-expressed CNPase (immature oligodendrocytes) and CC1 (more mature oligodendrocytes) (Figure 1F and 1G). We did not detect any double GFAP+/OLIG2+, GFAP+/SOX10+, GFAP+/DCX+, or DCX+/SOX10+ cells at any time. This sequence of events indicated successful labeling of SEZ NSCs and of their progeny. To further confirm this, SEZs were isolated 1 month post tamoxifen (n = 4) and neurospheres were generated. Upon induction of differentiation, we observed EYFP+ astrocytes (50.28% ± 14.61% GFAP+ cells), neurons (3.09% ± 2.65% beta-III-tubulin+ cells), and oligodendroglial lineage cells of different levels of maturity (most abundant were OPCs: 6.59% ± 2.76% NG2+ cells) (Figures 2A–2D).

### sezOPCs Transiently Populate the CC

**Figure 3:**
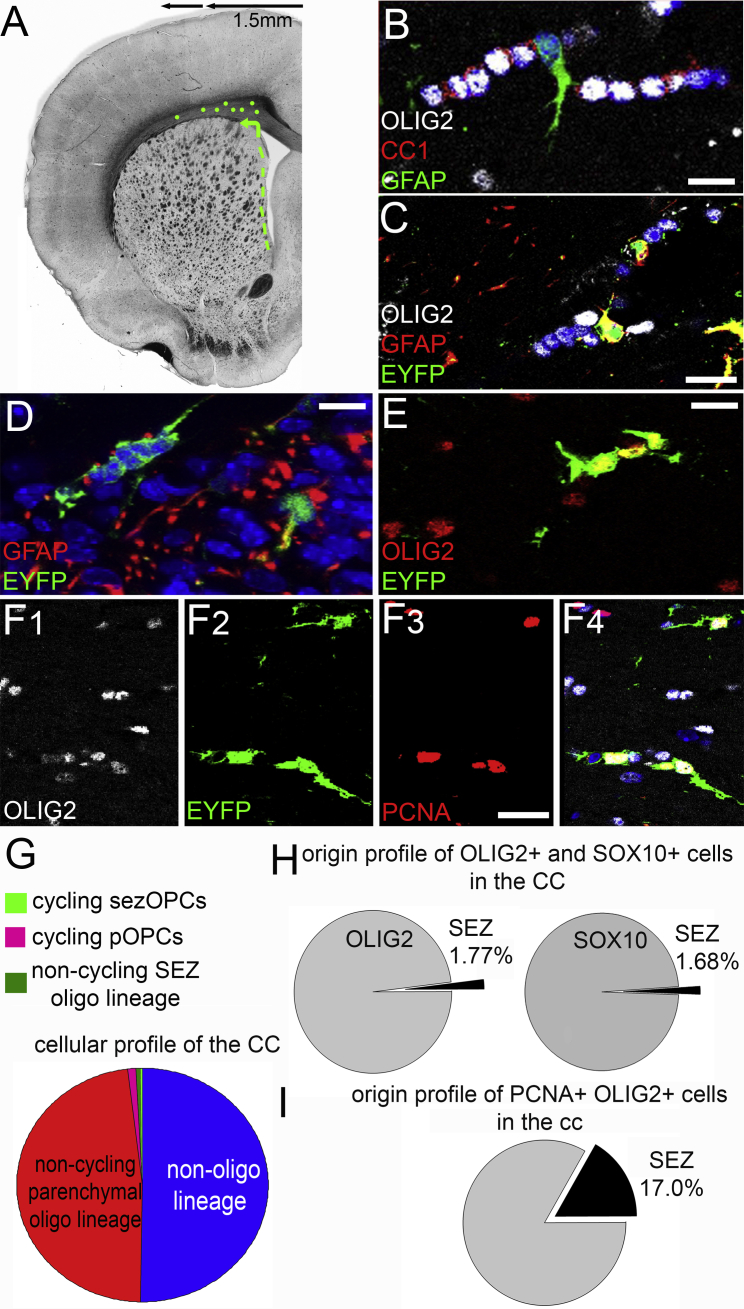
Contribution of SEZ Cells in the Intact Young Adult CC (A) Schematic illustration showing the distribution of EYFP+/OLIG2+ cells (green dots; the SEZ is highlighted by the dotted green line) within the supraventricular CC. (B) High magnification of characteristic chains of oligodendrocytes (OLIG2+/CC1+) in the CC with intercalated GFAP+ astrocytes. Note the OLIG2+/CC1− OPCs outside the chains. (C) Similar chains of cells in tamoxifen-treated mice with GFAP+ astrocytes co-expressing EYFP. (D and E) Clusters of EYFP+ cells in the CC. (F) Triple EYFP+/OLIG2+/PCNA+ cells in the CC. (G) Graph showing the profile of cells in the CC (n = 6 mice). Half of the cells belong to the oligodendroglial lineage; the majority are non-cycling OLIG2+ that do not express EYFP (red slice) while cycling pOPCs (pink), non-cycling SEZ-derived OLIG2+ (dark green), and cycling SEZ-derived cells (light green) constitute smaller fractions. (H and I) Graphs showing the contribution of SEZ-derived and parenchymal cells in the total pool and in the pool of dividing OPCs in the CC. Scale bars, 20 μm.

**Figure 4:**
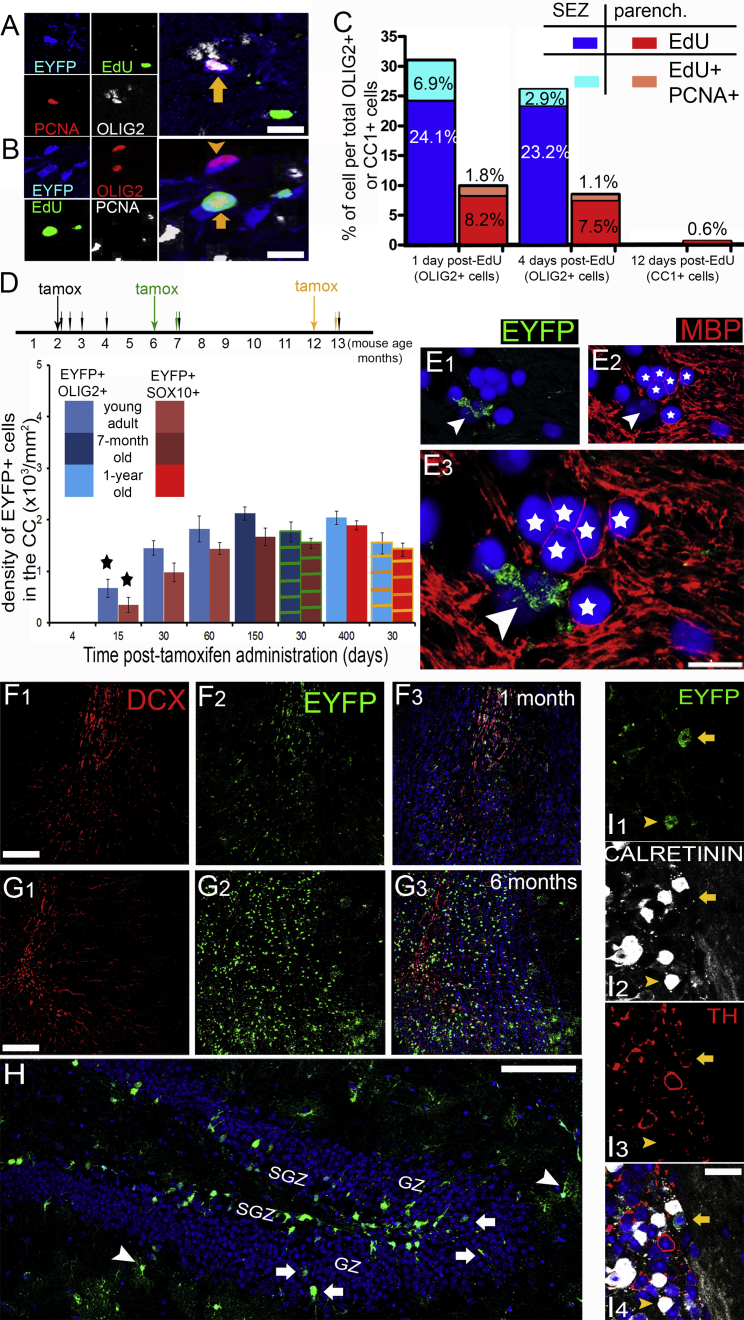
No Accumulation of Niche-Derived Cells in the CC (A and B) High-magnification image from the CC after quadruple staining for EdU, PCNA, OLIG2, and EYFP. The arrow in (A) indicates an EdU-negative mitotic SEZ-derived cell. The arrow in (B) indicates an EdU+ non-mitotic SEZ-derived cell. A non-mitotic, SEZ-derived cell is indicated by the arrowhead. (C) Graph showing the percentages of EdU+ and double EdU+/PCNA+ or EdU+/CC1+ cells within either the niche-derived or the parenchymal oligodendroglial lineage cell populations at 1, 4, and 12 days post-EdU administration (n = 4 mice per time point). Higher fractions of SEZ-derived cells are EdU+ and EdU+/PCNA+ at 1 and 4 days, but no EdU+ SEZ-derived cells were detected at 12 days (graphs show percentages of cells pooled by n = 3 mice per time point). (D) Graph showing the density of SEZ-derived OLIG2+ and SOX10+ cells in the CC at different times post-tamoxifen administration. The schema above explains the pulse/chase strategy (error bars = SD) (^∗^p < 0.05 compared with other time points, using one-way ANOVA and Scheffe post hoc analysis). Tamoxifen was administered to 2-, 6-, and 12-month-old mice (black, green, and yellow arrows, respectively) and animals were killed at different time points after tamoxifen (small arrows) (n = 4 mice per group for tamoxifen given at 2 months, n = 3 mice for the 6 and 12 month groups). The color of arrows and of lines on bars indicates the time point of tamoxifen administration, as described above. (E) High-magnification microphotograph of the CC showing a group of MBP+ cells (stars), near EYFP+/MBP− cells (arrowhead). (F and G) Microphotographs of immunostained olfactory bulbs 1 (F) and 5 (G) months post tamoxifen. Note the significant accumulation of EYFP+ cells over time. (H) Microphotograph of the dentate gyrus in a mouse 5 months post tamoxifen. EYFP+ cells are found deep within the granule neuron layer (GZ, arrows). Arrowheads show typical EYFP+ astrocytes (SGZ: subgranular zone neurogenic niche). (I) High-magnification microphotograph of the periglomerular layer in the olfactory bulb of a mouse 5 months post tamoxifen, immunostained for markers of mature interneurons, such as calretinin (white) and tyrosine hydroxylase (red). Note the presence of an EYFP+/TH+ cell (yellow arrow) and of an EYFP+/calretinin+ cell (yellow arrowhead). Scale bars, 10 μm (A, B, E); 50 μm (F, G); 100 μm (H); 20 μm (I).

SEZ-derived cells of the oligodendroglial lineage (expressing EYFP and OLIG2) were detected only within the supraventricular fragment of the CC, with 85.30% of all cells identified throughout the current study located within 1.50 mm from the midline of the brain and none detected beyond 2.00 mm (Figure 3A). None were identified on the septal side, the cerebral cortex, or the striatum, indicating that sezOPCs migrate to the CC through the dorsal part of the SEZ (Cayre et al., 2013). The CC architecture was characterized by chains of cells formed by OLIG2+/CC1+ oligodendrocytes, with few intercalated GFAP+ astrocytes (Figures 3B and 3C), while additional single OPCs (OLIG2+/CC1−) and astrocytes were found scattered throughout the tissue. We calculated the contribution of SEZ-generated cells in the total population of oligodendroglial lineage cells in the supraventricular CC in 3-month-old mice, 30 days after the administration of tamoxifen (n = 6). We distinguished OPCs (OLIG2+ or SOX10+/CC1−) from oligodendrocytes (CC1+) and identified cells of niche origin as EYFP+. Less than half of all CC cells expressed *Olig2* or *Sox10* (46.64% ± 10.35% and 39.07% ± 6.87%, respectively) with only 1.77% ± 0.12% of all OLIG2+ and 1.68% ± 0.34% of all SOX10+ cells co-expressing EYFP (EYFP+OLIG2+: 0.90% ± 0.01% of the total cell population, or 67 cells of a total of 3,789 OLIG2+ cells counted; SOX10+EYFP+: 0.73% ± 0.08% of the total cell population, or 60 of a total of 3,670 SOX10+ cells counted) (Figures 3G and 3H). Only 5.04% of all OLIG2+ cells co-expressed the proliferation marker proliferating cell nuclear antigen (PCNA). Although 4.25% of pOPCs were proliferating at any time, within the sezOPC pool this fraction was significantly higher at 29.16% (66 EYFP+/OLIG2+/PCNA+ cells out of a total of 228 EYFP+/OLIG2+ cells counted). As a result, in the CC, the contribution of sezOPC to the pool of cycling OPCs is higher than their contribution to the total pool of OPCs (approximately 1 in every 5 cycling OPCs versus only 1 in 45 of all OPCs) (Figures 3F and 3I). This difference in the proliferation profile between sezOPCs and EYFP−OPCs was confirmed in two additional ways. First, we co-immunostained brain tissue collected 1 and 4 days after the administration of ethynyl deoxyuridine (EdU) (n = 3 per time point, 30 days post tamoxifen administration) for EdU, EYFP, OLIG2, and PCNA. Significantly more sezOLIG2+ cells were positive for EdU or double-positive for EdU and PCNA, the latter having already divided once and undregoing a subsequent cell division (Figures 4A–4C). Second, we compared the mitotic activity of the two oligodendroglial progenitor pools by infusing the antimitotic drug cytosine β-D-arabinofuranoside (AraC) (or saline) at the surface of the brain for 4 days in order to ablate actively dividing cells in cortical and subcortical areas (n = 3 mice per group, 30 days post tamoxifen administration). The effectiveness of AraC was confirmed by the depletion of PCNA+ and DCX+ cells in the SEZ (Figure S3). Two days later, the numbers of PCNA+ cells were at normal levels while neuroblasts had just started to reappear; at 6 days post AraC proliferation had returned to control levels (Figure S3). When we measured the levels of OPC ablation in the CC at 2 days post AraC treatment we found that the density of EYFP−OLIG2+CC1− cells was unaffected ([48 ± 2.4] × 10^3^ cells/mm^3^, with a proliferation fraction of 3.83% ± 0.65% versus [53 ± 3.6] × 10^3^ cells/mm^3^, and a proliferation fraction of 4.25% ± 0.59% in the normal CC). In contrast, the density of EYFP+OLIG2+CC1− cells was significantly decreased ([1.2 ± 0.4] × 10^3^ cells/mm^3^, with a proliferating fraction of 5.56% ± 0.33% versus [1.8 ± 0.3] × 10^3^ cells/mm^3^, and a proliferating fraction of 21.66% ± 2.7% in the normal CC, p < 0.05 using Student's t test).

Based on the evidence that sezOPCs migrate and remain mitotic in the CC, and that they progress within the oligodendroglial lineage (expressing CC1), we hypothesized that SEZ-derived oligodendroglial lineage cells would accumulate in the supraventricular CC and came to dominate the local pool of OPCs and oligodendrocytes over time. We therefore investigated the number of EYFP+ cells of oligodendroglial lineage in the CC at different time points up to 13 months post tamoxifen. Our analysis revealed a plateau in the presence of EYFP+ cells at approximately 1 month after the initiation of labeling (Figure 4D). To confirm that sezOPCs were still generated in older mice and we were not observing an initial EYFP-retaining population, we administered tamoxifen in 6- and 12-month-old mice and collected tissue 1 month later. Numbers of EYFP+/OLIG2+ cells (total OPCs and oligodendrocytes) were similar in the 7- and 13-month-old mice, irrespective of whether the chase period was 1, 5, or 12 months (Figure 4D). From these data, we inferred a constant renewal of the sezOPC population and a failure in generation of long-lasting, terminally differentiated myelinating oligodendrocytes that would normally accumulate. To verify this, we used a longer chase of EdU in which immature oligodendrocytes generated at the time of EdU administration would exit from the cell cycle and retain EdU labeling. Although 0.6% of the parenchymal (EYFP−) CC1+ cells were found to retain EdU 12 days after administration (14 EdU+/CC1+ cells out of a total of 2,235 CC1+ cells), none of the niche-generated oligodendrocytes (EYFP+/CC1+ cells, in total 340 cells counted) were EdU+ (Figure 4C). Finally, when we immunostained for MBP, a marker of mature oligodendrocytes that labels both cell bodies and processes, and thoroughly analyzed the CC by confocal microscopy, we were unable to find any double EYFP+/MBP+ cells, in contrast to EYFP−/MBP+ cells (Figure 4E). To rule out the possibility that EYFP expression might be downregulated over time we immunostained olfactory bulb sections for EYFP, 1 and 5 months post tamoxifen. Labeled cells accumulated, with EYFP+ cells at the periglomerular layers expressing markers of interneurons such as calretinin and tyrosine hydroxylase (De Marchis et al., 2007) (Figures 4F, 4G, and 4I). EYFP+ cells could also be detected deep in the granular layer of the dentate gyrus, in which new granule neurons are generated from GFAP+ adult NSCs located in the subgranular zone (Figure 4H). Together, our results indicate that during adult brain homeostasis, waves of SEZ-derived cells of the oligodendroglial lineage migrate at the proximal region of the CC, where they remain mitotically active, are incorporated in the local cellular micro-structures, and express markers of maturation (CC1, CNPase), but ultimately fail to fully differentiate and are eventually lost.

### Niche and Parenchymal Oligodendrogenic Machineries Respond Differently to Demyelination

**Figure 5:**
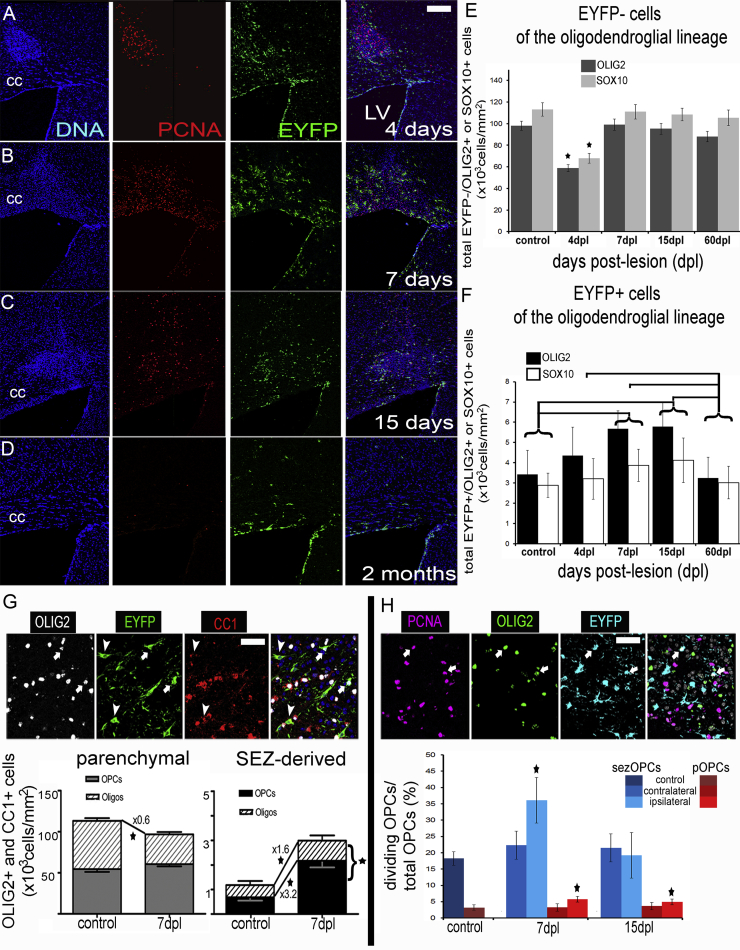
Dynamics of Oligodendroglial Lineage Cells after Demyelination in 2-Month-Old Mice (A–D) Tissue sections immunostained for EYFP and PCNA at 4 (A), 7 (B), 15 (C), and 60 (D) days post lesion. Note the absence of any visible signs of lesion at 2 mpl. (E) Graph showing the density of parenchymal OLIG2+ and SOX10+ cells after the lesion (^∗^p < 0.05, compared with the other time points and controls using one-way ANOVA, followed by the Scheffe post hoc analysis; error bars, SD). (F) Graph showing the density of SEZ-derived OLIG2+ and SOX10+ cells after the lesion (the horizontal bars show significant p < 0.05 changes between time points and controls using one-way ANOVA, followed by the Scheffe post hoc analysis; error bars, SD). (G) Representative image of the CC after immunostaining for OLIG2, EYFP, and CC1. Note the presence of EYFP+/CC1+ oligodendrocytes (arrowheads) and of EYFP+/OLIG2+/CC1− progenitors (arrows). The graph at the bottom left of the panel shows the density of parenchymal OPCs and oligodendrocytes in controls and at 7 dpl. The graph at the bottom right shows the respective information for the SEZ-derived cells of the CC. Overall numbers of sezOLIG2+ cells are significantly increased (indicated by the bracket), with both progenitors and oligodendrocytes being significantly increased (^∗^p < 0.05, using Student's t test; error bars, SEM). (H) Indicative image of the CC after immunostaining for PCNA, OLIG2, and EYFP. Note the presence of EYFP+/PCNA+/OLIG2+ cycling progenitors (arrows). The graph at the bottom shows the fraction of each population of OPCs that is proliferating in control mice and after demyelination (^∗^p < 0.05, compared with control levels using one-way ANOVA per OPC pool; error bars, SD. scale bars, 15 μm (G, H); 200 μm (A–D). n = 6 mice per time point).

The rate of oligodendrocyte turnover in the CC during homeostasis is low, but migration and proliferation of OPCs occur in response to focal demyelination. To challenge sezOPCs and pOPCs, lysolecithin was injected in the supraventricular CC 30 days after administration of tamoxifen inducing focal disruption of the tissue and demyelination; the lesion was fully resolved by 2 months (Figures 5A–5D). At 4 days post lesion (dpl) the total number of EYFP− oligodendroglial lineage cells was significantly decreased in the lesion (Figure 5E and data not shown). At 7 dpl the number of oligodendrocytes was still significantly depleted, but the total number of EYFP−/OLIG2+ cells had returned to normal levels (Figures 5E and 5G). pOPCs exhibited a significantly enhanced proliferative activity at both 7 and 15 dpl (Figure 5H). In contrast, numbers of EYFP+/OLIG2+ cells rose in response to demyelination and reached significantly higher than normal levels at 7 and 15 dpl (Figure 5F). At 7 dpl, SEZ-derived cells of the oligodendroglial lineage were significantly increased compared with controls: sezOPCs by approximately 3 times and cells expressing CC1 by 1.6 times (Figure 5G). The fraction of proliferating sezOPCs was significantly increased at 7 dpl, but returned to normal levels by 15 dpl (Figure 5H, black bars). To control for the possible contribution of callosal astrocytes to the detected EYFP+/OLIG2+ cell population, since astrocytes have been reported to transiently express *Olig2* after injury (Buffo et al., 2005), we infused OH-tamoxifen (100 μM, 2 μL) directly into the CC to label local astroglia, at the co-ordinates of the lesion 1 month before the focal demyelination and culled the mice (n = 4) at 7 dpl. Out of the 105 EYFP+ cells we observed at the area of lesion using confocal microscopy, none co-expressed OLIG2 (Figure S4), even those undergoing cell division, suggesting that astrocytic expression of OLIG2 was not a confounding factor in our labeling strategy.

Despite the increase in their numbers at 7 and 15 dpl, after full recovery at 2 months post lesion (mpl) the number of EYFP+ oligodendroglial cells had returned to normal levels (Figure 5F) and no EYFP+ myelinating oligodendrocytes could be detected. To exclude the possibility of an inherent inability of EYFP+ NSCs to progress in the lineage in vivo, in contrast to the in vitro assays (Figure 2), neurosphere cells generated from adult tamoxifen-treated mice were grafted into the CC at a time when myelination is in progress (21 days old, n = 3) and were culled 15 days later. We found 5.6% ± 1.3% of EYFP+ cells to express CC1 (17 cells out of 303) and 1.5% ± 0.4% to express MBP (6 cells out of 400 counted, one example shown in Figure 2E), while the rest co-expressed GFAP. No undifferentiated sezOPCs (EYFP+/OLIG2+) were detected. Despite the small numbers, these data indicate both that sezOPCs retain the capacity to generate MBP+ oligodendrocytes in vivo and that EYFP+ myelin can be detected. To test these conclusions, we used an in vitro myelination assay. We co-cultured freshly isolated SEZ cells with rat dorsal root ganglion cells for 21 days and found that 15.7% ± 6.4% of EYFP+ cells co-expressed MBP and exhibited the distinctive morphology of myelinating cells. High levels of EYFP expression were maintained especially in the cell bodies of mature oligodendrocytes (Figures 2F and 2G).

### sezOPCs Do Not Divide in the Aging CC but Respond to Demyelination

**Figure 6:**
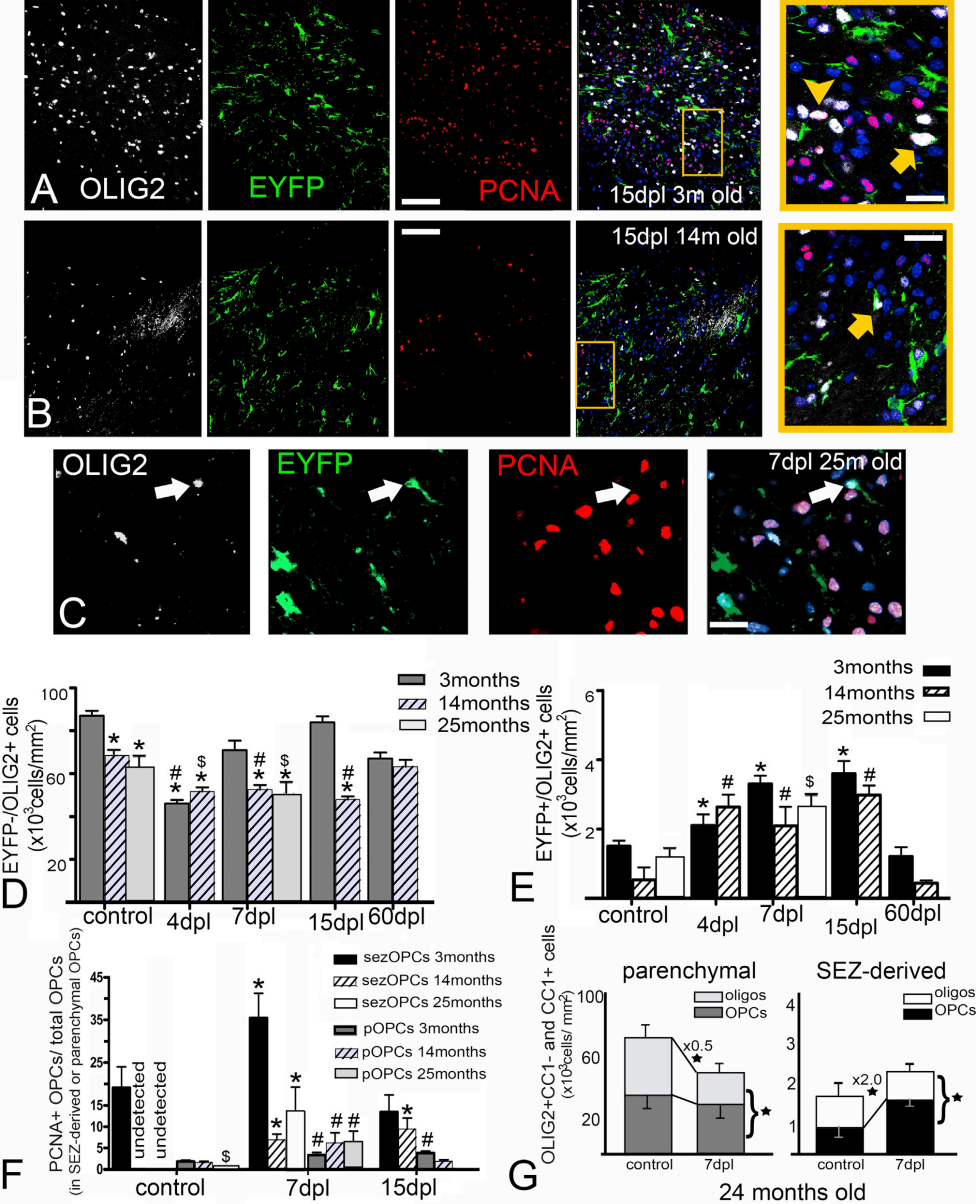
Dynamics of Cells of the Oligodendroglial Lineage after Demyelination in the Aging CC (A and B) Microphotographs of the CC from young (A) and aging (B) mice at 15 dpl after immunostaining for OLIG2, EYFP, and PCNA; a dividing pOPC and a dividing sezOPC are shown in the blown-up rectangle at the right end of (A), with an arrowhead and an arrow, respectively. A non-dividing sezOPC is shown in the blown-up rectangle at the right end of (B) with an arrow. (C) Microphotographs of the CC from an aged mouse at 7 dpl after immunostaining for OLIG2, EYFP, and PCNA (a sezOPC is indicated by the arrow). (D) Graph showing the density of OLIG2+ cells of parenchymal origin in control mice of different ages and at different dpl in young and 14-month-old CC, as well as at 7 dpl in the 25-month-old CC (^∗^p < 0.05 compared with control levels of the young adult mice; ^#^p < 0.05 compared with control levels in the 14month-group; ^$^p < 0.05 compared with control levels in the 25 month group; two-way ANOVA, followed by the Scheffe post hoc analysis; error bars, SEM). (E) Graph showing the respective information for the SEZ-derived component of the CC (^∗^p < 0.05 compared with control levels of the young adult mice; ^#^p < 0.05 compared with control levels in the 14 month group; ^$^p < 0.05 compared with control levels in the 25 month group; two-way ANOVA followed by the Scheffe post hoc analysis). (F) Graph showing the fraction of each population of OPCs that is proliferating in control mice of different ages and after demyelination. Note the absence of proliferating sezOPCs in the 14 and 25 month CC and the significant decrease in proliferating pOPCs in the 25 month CC (^∗^p < 0.05 compared with control levels of the same age group in sezOPCs; ^#^p < 0.05 compared with control levels in the same age group in pOPCs; ^$^p < 0.05 compared with control levels in the young adult mice for pOPCs; two-way ANOVA followed by the Scheffe post hoc analysis). (G) Graphs showing the density of parenchymal and SEZ-derived OPCs and oligodendrocytes in controls and 7 dpl 24-month-old mice. At 7 dpl the overall parenchymal OLIG2+ cell numbers are significantly lower compared with control mice (indicated by the bracket). Numbers of pOPCs (OLIG2+/CC1−) are at normal levels, while oligodendrocytes of parenchymal origin (CC1+) are significantly depleted. The total number of SEZ-derived cells is significantly increased at 7 dpl (indicated by the brackets), with sezOPCs being significantly increased and SEZ-derived oligodendrocytes being at normal levels (^∗^p < 0.05, using Student's t test). Error bars depict the SEM. Scale bars, 50 μm (A), (B); 20 μm in magnifications and in (C). n = 6 mice per time point for young-adult and 14-month-old mice, n = 4 for 25-month-old mice.

To assess whether the contribution of the two oligodendrogenic machineries of the supraventricular CC changes during aging we analyzed tissue from 14 (n = 7) and 25 (n = 4) month old mice, 1 month post tamoxifen administration. In both age groups the cell density in the CC was similar to that in 2-month-old mice (160 × 10^3^ cells/mm^2^ in young, 200 × 10^3^ cells/mm^2^ in 1-year-old, and 160 × 10^3^ cells/mm^2^ in 2-year-old mice); however, numbers of EYFP−/OLIG2+ cells were significantly decreased by approximately 25% (Figure 6D). The proliferating fraction of OPCs was at young adult levels in the 14-month-old mice but was significantly decreased in the oldest animals (Figure 6F). By contrast, although the numbers of SEZ-derived cells of the oligodendroglial lineage even in 2-year-old mice remained at levels similar to those of 2-month-old animals, we could find no dividing sezOPCs within the aging and aged CC (Figures 6E and 6F, control bars). We next demyelinated the CC and examined the tissue 4, 7, 15, and 60 dpl in mice lesioned at 6 and 14 months of age and at 7 dpl in the 25-month-old group (Figures 6A–6C and data not shown). No differences were found in the SEZ-derived and local oligodendroglial responses between 3-month-old and 6-month-old mice (data not shown). In the 1 year group, lysolecithin injection induced focal lesions of similar characteristics to those in 2-month-old mice (Figures 6A and 6B), with full remyelination achieved by 2 mpl. The EYFP− pool of oligodendroglial lineage cells remained significantly depleted at 7 and 15 dpl (in contrast to having recovered at similar post-lesion survival times in 2-month-old mice), but eventually recovered by 2 mpl (Figure 6D). In the 2year-old group, depletion of EYFP− cells of the oligodendroglial lineage was similar to that observed in 1 year old mice (Figure 6D). In both ages EYFP− OPCs exhibited young adult levels of post-lesion proliferation (Figure 6F) and their numbers at 7 dpl were similar to those of EYFP− OPCs in 7 dpl young-adult mice (Figure 6G). However, the population of EYFP−/CC1+ cells was slower to recover at 7 dpl in the aged CC (reduced by 1.6 times in 3 month old mice and by 2.0 times in 25 month old mice; compare Figures 5G and 6G). The niche-derived oligodendrogenic response was similar to that observed in 2 month old levels with numbers of EYFP+/OLIG2+ cells significantly increased following demyelination, even at the 4 dpl stage (Figures 6E and 6G). Notably, even though the sezOPC pool was mitotically inactive in homeostatic aging and aged CC, it showed a robust mitotic response after demyelination (Figure 6F). However, similar to the 2 month old, we were unable to detect any long-lasting contribution of the niche-derived cells to the fully remyelinated CC (Figure 6E).

### CC Demyelination Induces Proliferation in the SEZ Only in the Aged Brain

**Figure 7:**
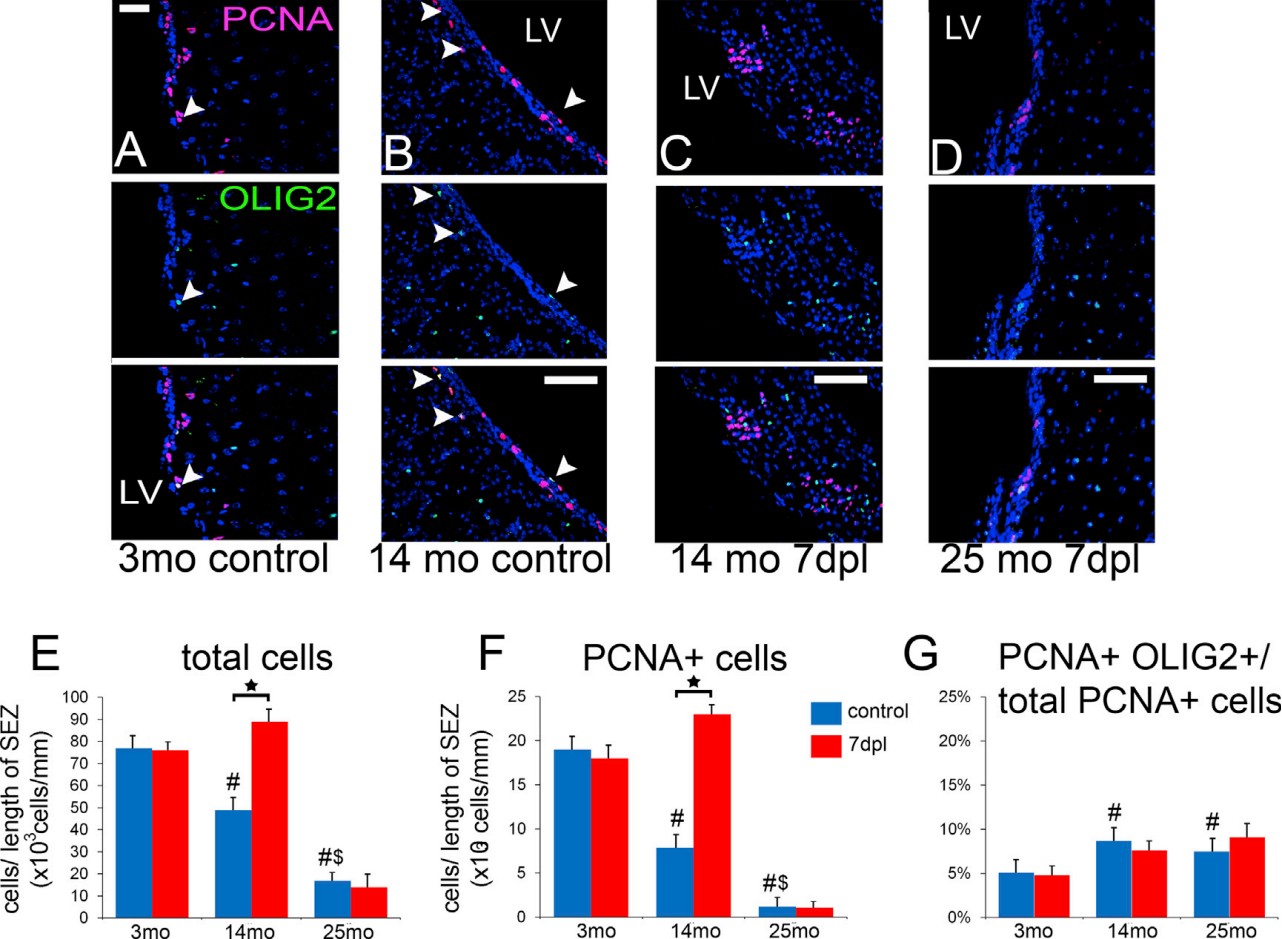
Dynamics of the Response of the SEZ after Demyelination in the CC (A–D) Microphotographs of the SEZ in young-adult (A), aging (B) and (C), and aged (D) mice, after immunostaining for PCNA and OLIG2. Note the increased occurrence of PCNA+/OLIG2+ cells in the homeostatic SEZ in the 14-month-old mice (double-positive cells are indicated with arrowheads). Also note the increased numbers of proliferating cells in the 14 month SEZ at 7 dpl (C), as well as the lower numbers of proliferating cells in the 25 month SEZ in (D). (E) The graph shows numbers of cells per length of the wall of the lateral ventricles in control and 7 dpl mice across different ages. (F) Graph showing numbers of PCNA+ cells in control and 7 dpl mice across different ages. (G) Graph showing the fraction of proliferating (PCNA+) cells that co-express OLIG2. Note the significant increase in oligodendrogenic mitoses in the aging and aged SEZ (^#^p < 0.05 compared with young-adult control levels; ^$^p < 0.05 compared with 14-month-old control levels; ^∗^p < 0.05 compared with control levels of the same age group; two-way ANOVA, followed by Scheffe's post hoc tests). Error bars depict SEM. Scale bars, 250 μm in (A–D). n = 6 mice per time point for young adult and 14-month-old mice, n = 4 for 25 month old mice.

The presence of increased numbers of sezOPCs in the area of demyelination at the early post-lesion stages could be explained either by the increased proliferation of local sezOPCs (already having migrated in the CC), or by an increased recruitment of cells directly from the cytogenic niche. We therefore investigated the response of the SEZ to demyelination in the 2 month old and the aging CC (Figure 7). We observed a significant reduction in the size of the SEZ (measured as density of cells) with aging: cell numbers being decreased approximately 2-fold in 14-month-old and 4-fold in 25-month-old mice (Figure 7E). Similar changes occurred within the proliferating cell population in the SEZ (Figure 7F); however, we observed a significant increase in the fraction of proliferating cells co-expressing *Olig2* over time (Figures 7A, 7B, and 7G), suggesting a tendency toward oligodendrogenesis in the aging SEZ. When the supraventricular CC was demyelinated in 2-month-old mice the SEZ remained unresponsive, with no changes in the numbers of proliferating cells or the lineage profile of proliferating cells (Figures 7F and 7G). In contrast, the 14-month-old cytogenic niche showed a robust response with a significant increase in the number of PCNA+ cells leading to the significant increase in the overall cell density of the SEZ (Figures 7B, 7C, 7E, and 7F), although we did not find any further switch in favor of OLIG2+ proliferating cells (Figure 7G). In the 25-month-old brain, the significantly depleted but still more oligodendrogenic SEZ failed to show any response to demyelination (Figures 7D and 7E–7G).

## Discussion

In the adult rodent brain, OPCs constitute a population of self-renewing cells that drive homeostatic myelin remodeling and post-injury remyelination (Franklin and ffrench-Constant, 2008, Young et al., 2013). Several studies have reported that adult NSCs in the SEZ generate OPCs that subsequently migrate to the CC and differentiate along the oligodendroglial pathway (Jablonska et al., 2010, Menn et al., 2006, Nait-Oumesmar et al., 1999, Ortega et al., 2013, Tong et al., 2015, Xing et al., 2014). Here, we investigated the properties of these two pools of OPCs during homeostasis and after demyelination in the young, aging, and aged brain. Our results generated three key conclusions: first, that SEZ-derived cells of the oligodendroglial lineage that migrate either to the intact or the focally demyelinated CC have limited migratory and self-renewal capacity. Second, that in either case they fail to generate mature myelin and, third, that these properties do not change significantly with age.

An earlier study reported that SEZ-derived progenitors account for less than 0.3% of total OPCs in the CC (Rivers et al., 2008). Here, we expand on this by demonstrating that SEZ-born cells of oligodendroglial lineage constitute at any time less than 2% of the total OPC population in the CC proximal to the lateral ventricles, even though they become incorporated into established cellular structures and start their differentiation program. Because new sezOPCs are continuously generated in the SEZ, as we have demonstrated by inducing tamoxifen-driven recombination at several ages (Figure 4D), if they had the capacity to self-renew, as pOPCs do, they would gradually accumulate within the tissue (Agathou et al., 2013). This is something that we did not observe by looking in numbers of EYFP+/OLIG2+ and EYFP+/SOX10+ cells over a period of 1 year. This lack of sustained self-renewing capacity is compatible with the properties of neuroblasts, the most committed progenitor type of the neurogenic output of the SEZ that are continuously generated by transit-amplifying progenitors only to migrate to their target area (the olfactory bulbs) where they have restricted proliferative activity and either differentiate or die (Lois and Alvarez-Buylla, 1994). Therefore, we propose that in the related system of SEZ-driven oligodendrogenesis the bona fide, self-renewing, OPCs remain stationary within the SEZ, being either the relatively quiescent NSCs (Ortega et al., 2013), or an oligodendrocyte-committed pool of transit-amplifying progenitors (Hack et al., 2005). Consequently, SEZ-derived cells of oligodendroglial lineage should be called oligodendroblasts and are not directly comparable with pOPCs. The transient nature of these cells seems to be confirmed by previously published reports in which SEZ-derived cells of oligodendroglial lineage expressing progenitor markers are only initially found in the CC after demyelination, being gradually replaced by more mature cells (Jablonska et al., 2010, Xing et al., 2014). Our results also invite caution when observing strong oligodendrogenic responses in and near the SEZ shortly after demyelination (Cate et al., 2010, Etxeberria et al., 2010), as these might not be translated to functional long-term oligodendrogenesis in the CC.

The transient presence of oligodendroblasts could also be explained by their progress into full maturation. Intriguingly, we failed to detect fully mature oligodendrocytes generated by oligodendroblasts. This was determined in three ways: first, by the absence of double EYFP+/MBP+ cells; second, by the lack of accumulation of EYFP+ cells in the CC, as would be expected if post-mitotic cells were constantly generated (a phenomenon we observed with neuroblasts differentiating in olfactory bulb interneurons). These results might be confounded by a gradual switch-off of EYFP expression during cell differentiation; however, we observed that EYFP expression is retained long-term in OB interneurons as well as in oligodendrocytes generated in vitro. In a third approach we also failed to detect EdU-retaining EYFP+ cells in the CC at 12 days post EdU administration. This observation could be due to EdU-induced toxicity on proliferating cells of SEZ origin (Ponti et al., 2013). Although we failed to detect such an effect in the pools of PCNA+, OLIG2+, or SOX2+ cells in the SEZ and the CC at 4 days post EdU (Supplemental Experimental Procedures, Table S2), we cannot exclude the possibility that the small population of oligodendroblasts within the CC might be specifically vulnerable to EdU toxicity, especially since they undergo more rounds of division compared with pOPCs (Figure 4C). Notably, we found that adult NSCs retain their inherent capacity to generate mature, MBP+ oligodendrocytes. This was revealed by co-culturing SEZ-derived neural stem and progenitor cells with dorsal root ganglion neurons, as well as by grafting them in the CC of 21 day old mice, at a time when progenitors of the dorsal SEZ contribute to CC oligodendrogenesis (Tong et al., 2015). Our results are consistent with a recent study in which whole-brain imaging and histological analysis failed to provide any evidence of SEZ-driven remyelination in the cuprizone model (Guglielmetti et al., 2014), but contradict another study in which high levels of remyelination were driven by oligodendroblasts after cuprizone-induced demyelination (Xing et al., 2014). It should be noted that in the latter study, in which demyelination was widespread, the contribution of niche-derived oligodendrogenesis in the area where we induced the focal lesion was low. Xing et al. suggest that sezOPCs might act in a bimodal way, with a first rapid-appearing wave reaching the area of the lesion without fully maturing into oligodendrocytes only to be supplemented by a second myelin-generating wave. Thus, in our model, where demyelination is more restricted and local pOPCs are less affected, we might be observing only the fast-response element of SEZ-driven oligodendrogenesis (see an example of relatively increased representation of oligodendroblasts in the core of the lesion in Figure S2F) because the second wave is not necessary. In the absence of differentiation the alternative fate of oligodendroblasts must be cell death. We failed to detect caspase-3+ (apoptotic) OLIG2+ cells either in the homeostatic or the post injury CC (data not shown), but low numbers of traced cells and fast removal of debris might have been a limiting factor, as apoptotic progenitors can be detected in the cell-dense SEZ (Kazanis et al., 2015). There is a third possibility that SEZ-derived progenitors eventually exit the CC and change their fate, but our labeling strategy would not allow for such a process to be observed.

The third major aim of this study was to investigate the relative effects of aging to SEZ-driven and parenchymal oligodendrogenesis. In the aging CC we found a significant decrease in total OLIG2+ cells, but the density of oligodendroblasts remained at young levels even at 25 months. The percentage of proliferating pOPCs was maintained to young-adult levels until 1 year and was significantly reduced in the 2 year CC. Strikingly, no proliferation could be detected in CC oligodendroblasts, although their fast-response properties after demyelination remained similar to that observed in the young CC; thus, their numbers were probably maintained by the switch toward oligodendrogenesis that we observed in the aging SEZ. In the 14 month old mice demyelinated CC, oligodendroblast numbers were boosted by a dramatic increase in both their proliferation locally, and in their generation in the SEZ. In the 25 month mouse brain, the proliferative capacity of the SEZ was reduced but the trend toward oligodendrogenesis remained, as has been recently demonstrated (Capilla-Gonzalez et al., 2013). Moreover, the levels of EYFP expression remained unchanged despite the reduction in cell density (Supplemental Experimental Procedures), and mitotic response in the CC was increased after demyelination.

Overall, our data support the view that the SEZ is a source of plasticity for the adult CC, and that oligodendroblasts could be a valid therapeutic tool for demyelinating diseases if their generation from wider parts of the SEZ could be stimulated, if their response could be sustained for long periods, and if the reasons that lead to variable levels of remyelination capacity between different models of demyelination are elucidated. They also complement recently reported evidence that different pools of OPCs contribute in discrete and specific ways to remyelination (Crawford et al., 2016). Finally, they highlight the fact that pOPCs manifest an advantage in generating oligodendrocytes when compared with SEZ oligodendrogenesis and that this does not change in the aging brain.

## Experimental Procedures

### Animals and Administration of Tamoxifen

Adult C57/BL6 mice were used, generated from the crossing of hGFAP-Cre^Ert2^ and Rosa26-EYFP transgenic mice (Hirrlinger et al., 2006) that were homozygous for both transgenes. Animal breeding, maintenance and experimental procedures were conducted in accordance with the UK Animals (Scientific Procedures) Act 1986, authorized by the Home Office and scrutinized by the Animal Welfare and Ethical Review Body of the University of Cambridge. The male:female ratio was kept at 1:1. For the induction of cre-mediated recombination, tamoxifen was injected intraperitoneally once every day for six consecutive days with 2 mg (in 100 μL) per 30 g of body weight (stock solution: 20 mg/mL, in 90% corn oil, 10% ethanol). In all demyelination experiments tamoxifen was administered 1 month before the lesion. For the EYFP accumulation experiments, tamoxifen was given to 2-month-old mice (the young-adult age category) and tissue was collected at 4, 15, 30, 60, 150, and 400 days. In two groups of mice (n = 3 per group) tamoxifen was administered at 6 and 12 months of age, respectively, and tissue was collected after 30 days (Figure 4D, dark and light blue/red hues, respectively).

### Lysolecithin Injections and Infusion with AraC

To induce focal demyelination in the CC, mice received a unilateral injection of lysolecithin (1%, 2 μL injected with a Hamilton syringe at a rate of 1 μL/min) at 30 days post tamoxifen, under inhalation anesthesia (iso-fluorane). Mice were culled at 4, 7, 15, and 60 dpl. To ablate proliferating cells, animals (30 days after tamoxifen administration) were anesthetized and a cannula (BIK-II, ALZET) was fixed on the skull (1 mm lateral to bregma) connected to a subcutaneously implanted mini-osmotic pump (1007D, ALZET). AraC (4%; Sigma, C1768, or saline) was infused for 4 days onto the surface of the brain and animals were culled immediately after as well as at 2 and 6 days post infusion. Numbers of proliferating cells were counted within the SEZ in order to assess the efficiency of the infusions. Numbers of oligodendroglial cells were counted within the CC at 2 days post AraC. This time point was chosen in order to avoid the high levels of GFAP activation normally observed at early post-infusion stages and to limit the possibility that the results will be confounded by the transient loss of SEZ-derived cells that might be affecting the sezOPC pool at later time points.

### Tissue Processing, Immunostainings, EdU Assays, and Imaging

Animals were culled by transcardial infusion of 4% paraformaldehyde and tissue was post-fixed overnight in 2% paraformaldehyde (at 4°C). Sections were taken with a Leica cryostat (12 μm thick) and were processed for immunohistochemistry using various antibodies (see the Supplemental Experimental Procedures). For the short- and long-term EdU chases, EdU was injected intraperitoneally twice per day for 2 days (50 μg/g of body weight; 50 mg/mL) 30 days after tamoxifen administration, and mice were culled 1, 4, and 12 days after the second injection. Possible EdU-induced toxicity on oligodendroglial lineage and proliferating cells was assessed as described in the Supplemental Experimental Procedures and was found to be non-significant (Table S2). EdU was detected following the Click-iT EdU Imaging Kits protocol (Invitrogen, Molecular Probes). Images were acquired using a Leica SP5 confocal microscope and were processed using Photoshop (Adobe) software. For stereological analysis (structure of the aged niche and investigation of EdU-induced toxicity), optical layers were acquired at a 0.8 μm step and were then processed using the Imaris and Volocity software (see the Supplemental Experimental Procedures).

### Neurosphere Cultures and Grafts of Progenitors

To grow adult neural stem and progenitor cells in vitro, the SEZs of 3-month-old tamoxifen-administered mice were dissected, dissociated, and plated in growth medium (see the Supplemental Experimental Procedures). For the differentiation assays, fourth-passage neurospheres were dissociated and plated on poly-D-lysine-coated coverslips in the same medium without growth factors. For grafting experiments, second-passage neurosphere cells were dissociated in HEPES buffer and injected directly beneath the bregma in wild-type 21 day old mice, under inhalable anesthesia. Approximately 50,000 cells were injected in a total volume of 2 μL. Mice were culled 15 days later and their brains were perfused, fixed, and stained as described previously with the following antibody cocktails: OLIG2, EYFP, and MBP, or GFAP, EYFP, and CC1.

### Co-culture Myelination Assays

This culture system was established using protocols described previously (Lundgaard et al., 2013, Wang et al., 2007) (see also Supplemental Experimental Procedures). Briefly, dorsal root ganglia (DRGs) were dissected from embryonic day 14–16 rats, dissociated, and plated onto 22 mm coverslips coated with poly-D-lysine followed by growth factor-reduced Matrigel at a density of 5 × 10^5^cells/mL. Freshly isolated SEZ cells were generated as in neurosphere preparations, kept in culture (growth medium) for 3 days, and dissociated with Accutase (Millipore) on the day of plating on DRGs (100,000 cells were plated in each well).

### Measurements and Statistical Analysis

For the investigation of the homeostatic CC and SEZ, at least two sections from four rostrocaudal levels (+1.5, +1.0, +0.5, and −0.2 in respect to bregma) of the forebrain were immunostained and analyzed for EYFP, OLIG2, and CC1, or EYFP, OLIG2, and PCNA, respectively, at all ages. In 2-month-old mice the analysis was repeated by immunostaining for EYFP, SOX10, and GFAP. Co-expression of various molecules was assessed by marking positive cells independently for each immunostaining and subsequently stacking all markings together (see also the Supplemental Experimental Procedures). Statistical analyses were performed using Microsoft Office Excel or SPSS software. When comparing different post-lesion or post-tamoxifen administration time points, one-way ANOVA was used followed by post hoc tests. When comparing different post-lesion time points and ages, two-way ANOVA was used, followed by post hoc tests. When comparing sezOPCs and pOPCs for expression of markers or for densities, Student's t test analysis was used. Statistical significance was always set to p = 0.05.

## Author Contributions

I.K. designed and performed experiments, analyzed the data, and wrote the paper. K.E. performed experiments. E.A., C.D., and C.K. performed experiments and data analysis. R.T.K. contributed materials and to the writing of the paper. R.J.M.F. designed the experiments and wrote the paper.

## Supplemental Information

Document S1. Supplemental Experimental Procedure, Figures S1–S4, and Tables S1 and S2

Movie S1. 3D Reconstruction of SEZ-Derived OLIG+ Cells

Document S2. Article plus Supplemental Information
