# Supplementary material for: Subependymal Zone-Derived Oligodendroblasts Respond to Focal Demyelination but Fail to Generate Myelin in Young and Aged Mice
Source: Stem Cell Reports. 2017 Feb 9;8(3):685–700. doi: 10.1016/j.stemcr.2017.01.007 (PMC5355571; doi:10.1016/j.stemcr.2017.01.007)
Supplement: Document S1. Supplemental Experimental Procedure, Figures S1–S4, and Tables S1 and S2 [file mmc1.pdf]

Stem Cell Reports, Volume *8*

Supplemental Information

Subependymal Zone-Derived Oligodendroblasts Respond to Focal Demyelination but Fail to Generate Myelin in Young and Aged Mice

Ilias Kazanis, Kimberley A. Evans, Evangelia Andreopoulou, Christina Dimitriou, Christos Koutsakis, Ragnhildur Thora Karadottir, and Robin J.M. Franklin

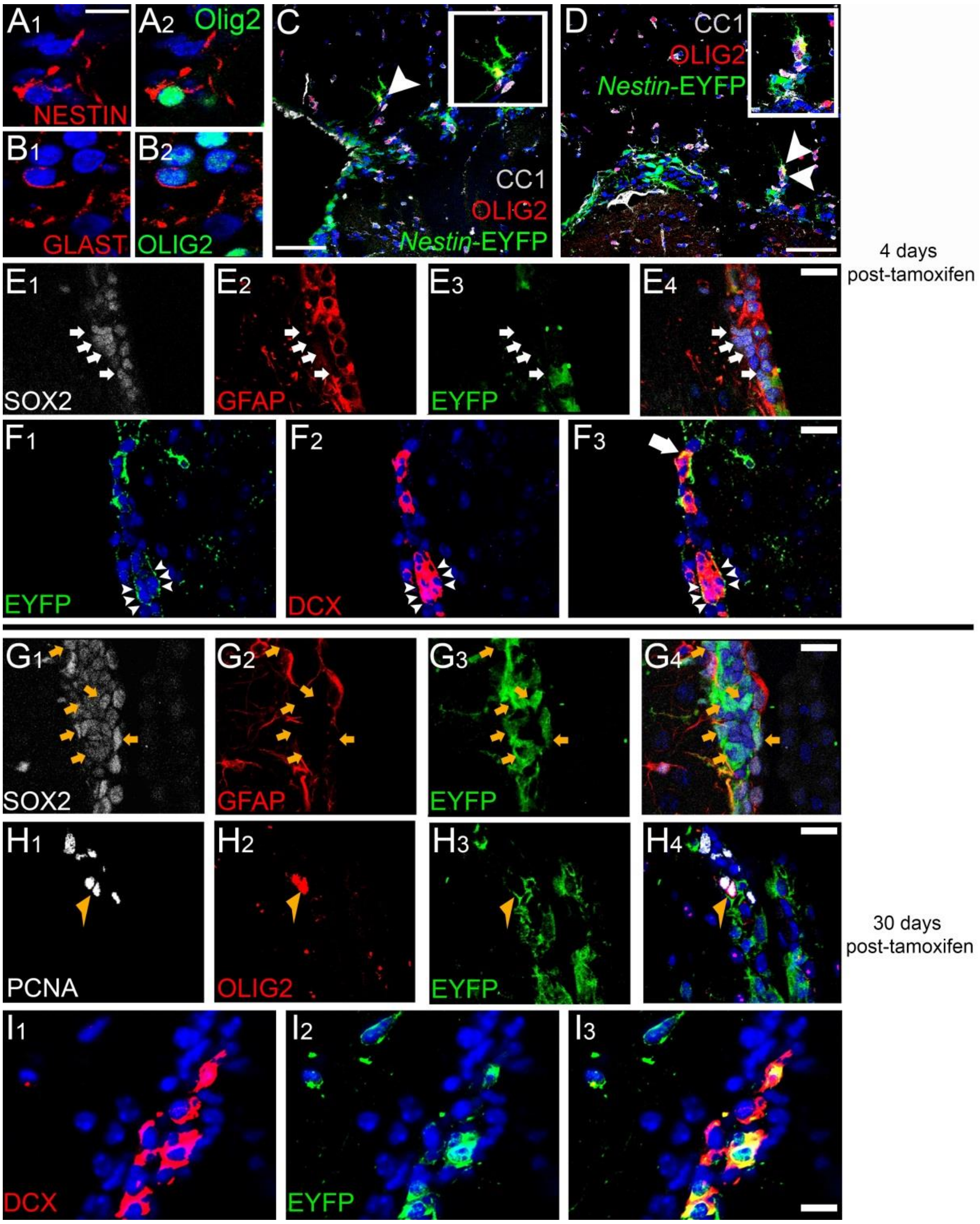

Figure S1

Assessment of labeling strategy [linked to Figure 1 and experimental procedures]

(Panels A,B) In the CC, expression of *Nestin* (A) and *Glast* (B) was observed in a fraction of OLIG2+ pOPCs. (Panels C,D) In the CC of nestin-Cre^{Ert2} x Rosa26-EYFP mice, four days after the end of tamoxifen administration, few EYFP+OLIG2+ cells (indicated by arrowheads and magnified in the insets) were observed. (Panels E,F) At 4 days post-tamoxifen EYFP expression was detected mainly in GFAP+ astrocytes and not in other progenitors, either SOX2+ (arrows in E), or DCX+ (in red in F, where arrowheads indicate EYFP+ astrocytic processes forming the glial tubes trhough which neuroblasts migrate. The big arrow indicates one DCX+ EYFP+ cell). (Panels G-I) At 30 days post-tamoxifen EYFP is expressed in multiple SOX2+ and GFAP- progenitors (yellow arrows in G), in mitotic OLIG2+ cells (yellow arrowhead in H) and in DCX+ neuroblasts (in I). [In panels E-I tissue is derived from hGFAP-Cre^{Ert2} x Rosa26-EYFP mice. Scale bars: A,B 10µm; C,D 50µm; E,F 30µm; G,H 20µm; I 10µm]

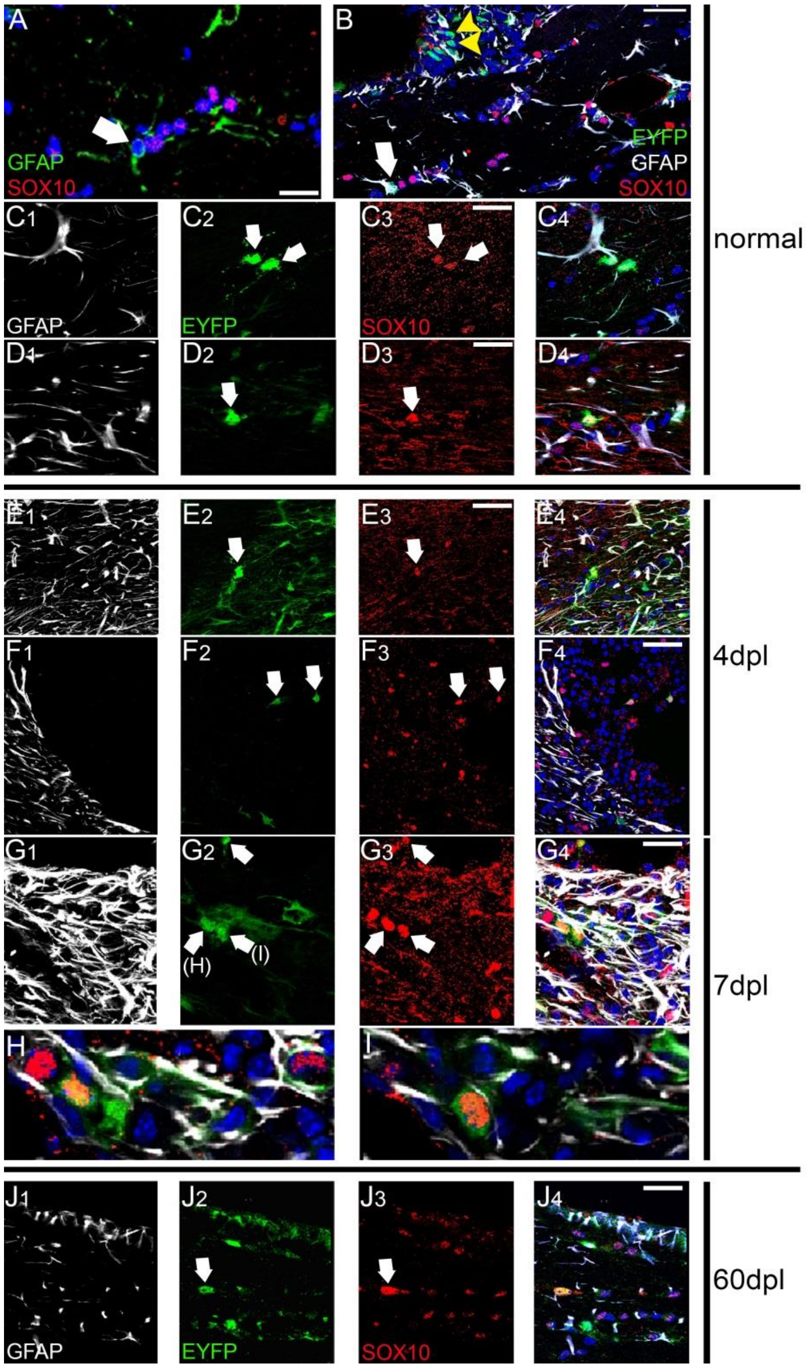

Figure S2
Expression of Sox10 in SEZ-derived cells of the CC [linked to Figures 3,4,5]

In the normal CC GFAP+ astrocytes (arrow in A) are intercalated in chains of SOX10+ oligodendroglial lineage cells. At 30 days post-tamoxifen (B) EYFP expression is observed in such intercalating astrocytes (arrow in B), but also in GFAP- cells in the SEZ (yellow arrowheads in B). Within the CC, EYFP+ cells that express *Sox*10 but not *Gfap* were detected (indicated by arrows in C,D). Within the area of lesion at 4dpl and at 7dpl multiple GFAP-SOX10+EYFP+ cells were detected (indicated by arrows in E,F,G). Note the presence of EYFP+SOX10+ cells in the astrocyte-poor core of the lesion at 4dpl (two arrows in F). The two adjacent EYFP+SOX10+GFAP- cells shown in the stack of confocal planes of panel G are shown in separate confocal planes in H and I. At 60dpl, when the area of lesion has been fully regenerated, the number of EYFP+SOX10+GFAP- cells (arrow in J) reverses to normal levels and the cytoarchtecture resembles that of the normal CC. [Scale bars: A,C,D,G 10μm; B,E,F,J 25μm]

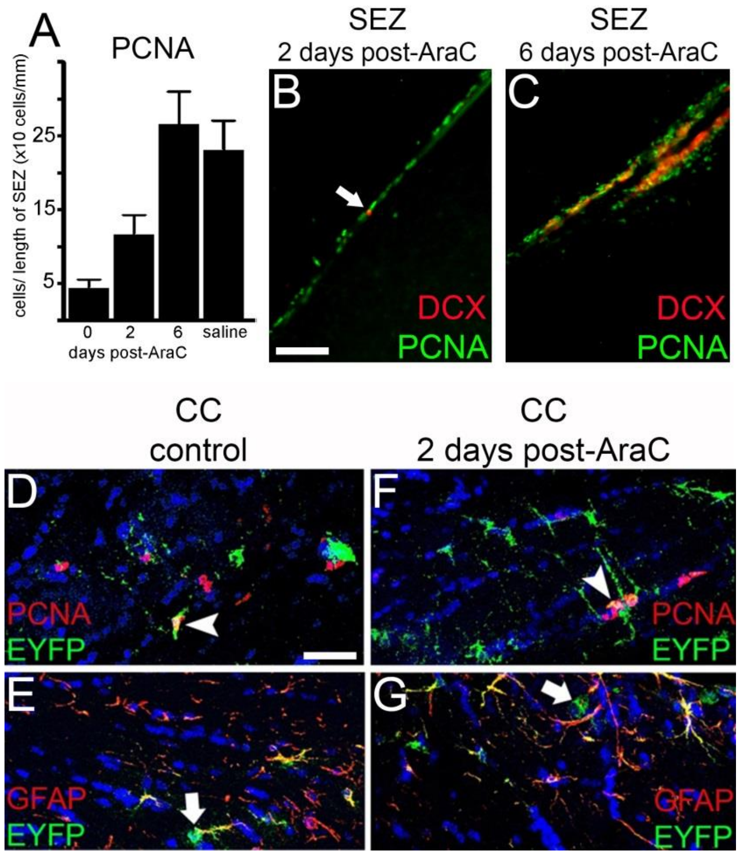

Figure S3
Effects of AraC infusion in the SEZ and the CC [linked to Figure 7 and Experimental procedures]

(A) Graph showing numbers of PCNA+ cells within the SEZ at different time-points after the end of AraC infusion. Note that proliferating cells are depleted by AraC, with their numbers gradually bouncing back until day 6 post-infusion. (n=3 mice per group, error bars: SEM) (B, C) Microphotographs of the SEZ after immunostaining for DCX (in red to mark neuroblasts) and PCNA (in green to mark dividing cells). Note that 2 days after the end of AraC infusion (B) multiple PCNA+ cells have re-emerged in the depleted SEZ. However, neuroblasts are only starting to appear (white arrow). Mitotic cells are either NSCs, or transit amplifying progenitors (type C cells). 4 days later (C) the numbers of neuroblasts have increased significantly. (D-G) Microphotographs of the saline and AraC infused CC after immunostaining for EYFP (in green) and PCNA (D, F) or GFAP (E, G). Note that the structure and cell profile of the CC is not significantly altered by AraC infusion with the exception of a mild activation of astrocytes (more processes were observed). White arrowheads in D and F indicate EYFP+PCNA+ cells. White arrows in E and G indicate GFAP-EYFP+ cells. [scale bars: 150μm B-C; 30μm D-G]

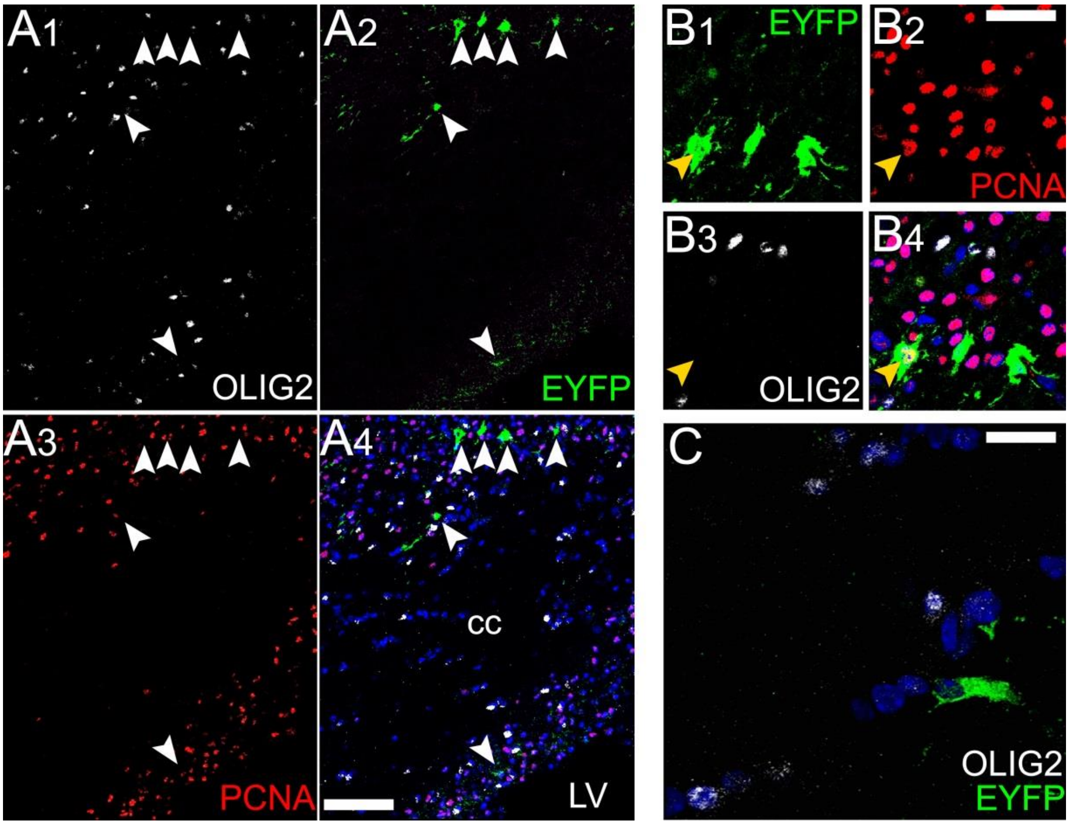

Figure S4

Lack of contribution of local astrocytes in OLIG2+ cells post-demyelination (linked to Figure 5]

OH-tamoxifen was injected directly into the CC, in order to drive EYFP expression in local astrocytes, and a focal demyelinating lesion was induced after one month. (Panels A, B) At 7dpl several EYFP+ cells were detected at the area of demyelination (white arrowheads in A). However, none of these cells was co-expressing OLIG2 (in white), even in conditions of mitosis (note one EYFP+ PCNA+ OLIG2- cell in B). In the homeostatic CC, one month post OH-tamoxifen injection, EYFP+ cells were all astrocytes often intercalated with OLIG2+ cells (panel C) [scale bars: A 50µm; B 25µm; C 10µm].

Legend to Movie S1 (linked to Figure 1)

3D reconstruction of SEZ-derived OLIG+ cells

3D reconstruction -in high magnification- of the corpus callosum, after having performed immunostaining for EYFP (in green) and OLIG2 (nuclear staining, in red) and counterstaining for DNA (Dapi fluorescence, in blue). Note two double positive cells that based on their morphology are most likely to be OPCs.

Table S1. *Distribution of different cell types within the SEZ and the CC in mice given tamoxifen at 2 months postnatally [linked to Figures 1, 3 and 4].*

	**Ependymal cells**	**GFAP+**	**SOX2+**	**DCX+**	**OLIG2+**	**SOX10+**	**PDGFRα+**
Total SEZ cell population (irrespective of EYFP expression)	22.25%±2.4	44.85%±3.5	85.48%±7.2	30.76%±4.2	3.12%±1.3	2.15%±0.4	1.56%±0.6
EYFP+ cells in the SEZ (4 days post-tamoxifen)	16.05%±2.5	79.96%±5.7	89.67%±9.2	4.00%±1.4	n.d.	n.d.	n.d.
EYFP+ cells in the SEZ (30 days post-tamoxifen)	7.15%±2.6	44.85%±5.4	82.65%±9.9	43.49%±6.3	2.43%±1.2	0.90%±0.2	0.67%±0.33
	**GFAP+**	**DCX+**	**OLIG2+**	**SOX10+**	**CC1+**	**CNPase+**	
EYFP+ cells in the CC (4 days post-tamoxifen)	92.23%±4.5	n.d.	n.d.	n.d.	n.d.	n.d.	
EYFP+ cells in the CC (30 days post-tamoxifen)	82.76%±6.3	0.67%±0.3	3.43%±1.2	2.89%±0.6	3.12%±2.1	1.32%±0.6	

The overall distribution of cell types in the SEZ (first row) was calculated by pooling the results generated from mice of both post-tamoxifen groups (4 and 30 days after the end of administration) looking at cells irrespective of EYFP expression. Note that at the early time-point no EYFP+ cells co-expressing oligodendroglial lineage markers could be detected and only a minimal fraction of DCX+ neuroblasts was observed. Within the CC the majority of EYFP+ cells was co-expressing GFAP, with other lineages appearing beyond the 4-day post-tamoxifen time-point. It should also be noted that we did not detect any cells co-expressing GFAP and either OLIG2, SOX10, or DCX.

Table S2. *Density of different cell types within the SEZ and the CC 4 days after the administration of EdU [linked to Figure 4 and Experimental Procedures]*.

		OLIG2 (x10^3cells/mm^2)	PCNA (x10^3cells/mm^2)	OLIG2;PCNA (x10^3cells/mm^2)	SOX2 (x10^3cells/mm^2)	PCNA;SOX2 (x10^3cells/mm^2)	fraction of OLIG2 that are PCNA+	fraction of PCNA+ that are OLIG2+
**SEZ**	-EdU	0.051±0.014	0.077±0.022	0.003±0.001	0.432±0.076	0.042±0.012	5.64%±3.23	2.97%±1.29
	+EdU	0.032±0.009	0.061±0.031	0.001±0.001	0.392±0.032	0.043±0.015	4.71%±2.01	3.24%±1.18
**CC**	-EdU	0.082±0.031	0.132±0.044	0.005±0.001	0.782±0.082	0.073±0.038	5.35%±2.02	3.15%±1.77
	+EdU	0.048±0.026	0.108±0.076	0.003±0.001	0.700±0.091	0.075±0.029	6.56%±3.18	4.97%±2.08

4 days after EdU administration the numbers of OLIG2+, SOX2+ and PCNA+ progenitors are not significantly different within the SEZ and the CC (using t-test analysis), although average cell density of OLIG2+ cells is lower. Numbers of proliferating OLIG2+ and SOX2+ cells and the proliferating fraction within the OLIG2+ pool are also normal after EdU administration.

Supplemental Experimental Procedures (linked to Figure 1 and experimental procedures)

Choice of labelling strategy and assessment of labelling efficiency

In order to decide and finalize our labelling strategy, we investigated the expression of *Glast* and *Nestin* in the CC in order to determine if these progenitor markers are expressed in a fraction of pOPCs. In our hands significant expression of both was detected in the supraventricular CC (16.4%±2.4 and 9.3%±3.1 of OLIG2+ cells in the CC co-expressed nestin and Glast, respectively; Figure S1A,B). Furthermore, sections from nestin-Cre^{Ert2} x Rosa26-EYFP mice were assessed one day after the end of tamoxifen administration and EYFP+ OLIG2+ cells were found in the CC (Figure S1C,D). Based on the above we decided not to use either the Glast-Cre^{Ert2} or the nestin-Cre^{Ert2} transgenic mice.

The suitability of the chosen transgenic system for labelling SEZ-driven cytogenesis was assessed by characterizing EYFP+ cells in the SEZ and our area of interest, the CC, at different time-points after administration of tamoxifen (Figure S1 and table S1). According to our analysis, the 2-month old SEZ contained ependymal cells (22.25%±2.4), astrocytes (44.85%±3.5), neuroblasts (30.76%±4.2) and oligodedroglial cells (alternative labelling with SOX10+: 2.15%±0.4; OLIG2+: 3.12%±1.3; PDGFRα+: 1.56%±0.6). At 4 days post-tamoxifen EYFP+ cells included ependymal cells (16.05%±2.5), astrocytes (79.96%±5.7) and neuroblasts (4.00%±1.4), with no oligodendroglial lineage cells. At 30 days post-tamoxifen the generation of neuroblasts and OPCs from the initially labelled NSCs (that are included in the astrocyte pool) changed the cell-type representation within the EYFP+ pool, that now included (as fraction) less ependymal cells (7.15%±2.6), less astrocytes (44.85%±5.4), but more neuroblasts (43.49%±6.3) and OPCs (SOX10+: 0.90%±0.2; OLIG2+: 2.43%±1.2; PDGFRα+: 0.67%±0.33). In the CC, at the 4-day time-point the vast majority of EYFP+ cells were GFAP+ (92.23%±4.5) and the rest were unidentified cells. No EYFP+ cells co-expressing OLIG2, SOX10, CC1 or DCX could be observed (PDGFRα immunostainings were very difficult to assess and quantify in the CC). At 30 days post-tamoxifen EYFP+ cells in the CC included GFAP+ cells (82.76%±6.3) and a GFAP- cell fraction that expressed DCX+ (0.67%±0.3 of all EYFP+ cells) or oligodendroglial lineage markers (SOX10+: 2.89%±0.6; OLIG2+: 3.43%±1.2; CC1+: 3.12%±2.1; CNPase+: 1.32%±0.6); the rest were unidentified cells. The efficiency of tamoxifen-induced recombination in the hGFAP-Cre^{Ert2} x Rosa26-EYFP mice was estimated based on the appearance of EYFP+ cells in three different cell populations 1 month post-administration. This

time-window was necessary because it was not possible to assess recombination specifically in NSCs, due to their similar morphology to non-stem cell astrocytes. Therefore, we had to wait for the major cytogenic output of NSCs (the neuroblasts) to be fully developed after administration of tamoxifen, assuming that labelling efficiency in NSCs would be translated in equal labelling efficiency in their progeny. Within the SEZ, on average, 43.4%±15.3 of GFAP+ cells were co-expressing EYFP. However, when the pool of DCX+ neuroblasts was analysed the percentage of EYFP+ cells was found to be on average 34.7%±11.1 in the SEZ and 37.3%±13.3 within the olfactory bulbs ($p<0.05$; using ANOVA). Interestingly, though, the fraction of EYFP+ neuroblasts was found to be significantly higher 5 months post-tamoxifen administration (in the SEZ 46.8%±12.7 of all DCX+ cells and 52.8%±19.4 of all GFAP+ cells co-expressed EYFP), indicating that the initial difference in the labelling efficiency between all astrocytes and NSCs (as reflected in the percentage of their EYFP+ progeny) was due to the slow cell-cycle kinetics of adult NSCs. In an alternative analysis, we expressed the volume of EYFP+ cell bodies and processes per the total volume of the SEZ using serial optical fields across the dorsoventral length of the SEZ in 2 month and 24 month-old mice that had received tamoxifen 1 month before, as well as in 24-month old mice that had received tamoxifen when they were 12-months old. The confocal generated *.lif files were first converted to the Imaris file format (*.ims) and were subsequently 3D-stitched using XuvTools 1.8.0 (Emmenlauer et al., 2009). To analyze the total EYFP in the stitched files, Imaris 7.4.2 (Bitplane AG) was utilized as follows: the EYFP channel was transformed into a 3D surface using a “smooth surfaces” parameter with an area detail level of 1μm and tweaking the threshold value accordingly to correspond to the actual fluorescence levels as close as possible. The detailed 3D surface creation parameters are mentioned in the table below. The total EYFP expression was then measured as the sum volume of the 3D surface, allowing comparison between the two samples. An additional measurement was performed by dividing the EYFP 3D surface sum volume by the “data volume” of each file (the total volume of the dataset), expressing the EYFP presence as a comparable percentage. Our analysis revealed no differences in the levels of EYFP expression in 24-month old mice 30 days post-tamoxifen, as compared both to 24-month old mice 1 year post-tamoxifen and to 2-month old mice (6.50% vs 4.32% and3.92%, respectively). However, due to the described significant reduction in the numbers of progenitors in 24-month old SEZ these results indicate a possible increase in the recombination efficiency, or in the presence of astrocytes in the aged SEZ. However, the analysis does not allow assessing if higher numbers of NSCs are present in the aged SEZ.

Lysolecithin injections

The stereotaxic co-ordinates of the site of injection were: 0.5mm rostral and 0.8mm lateral to bregma, with lysolecithin injected at 1.5mm below the dura. All mice recovered well from the procedure and were given analgesia (Vetergesic) prior to the lesion; analgesia was also given post-operatively if necessary. For the investigation of the contribution of local astrocytes to the pool of OLIG2+ cells post-demyelination, OH-tamoxifen (100nM) was injected directly into the CC and lysolecithin was injected one month later. Mice were killed 7dpl.

Tissue processing, immunostainings

Animals were killed by transcardial infusion of 4% paraformaldehyde and tissue was post-fixed overnight in 2% paraformaldehyde (at 4ºC). Brains were immersed in 30% sucrose for 48h (at 4ºC), embedded in gelatine/ sucrose (7.5%/ 15% w/v) and frozen at -50ºC in iso-pentane (Sigma-Aldrich, UK). For immunohistochemical stainings the following antibodies were used:
Chicken or goat anti-GFP (Abcam, 1/500, ab13970 and ab5450, respectively) was used to detect EYFP since levels of endogenous EYFP were under the detection threshold of the microscopes. Rabbit anti-OLIG2 (1/200, Millipore, AB9610), rabbit anti-NG2 (Abcam, 1/200, ab101808), rabbit anti-PDGFRα (Abcam, 1/200, ab51875) and goat anti-SOX10 (SantaCruz, 1/100, sc-17342) were used to identify OPCs. Mouse anti-CC1 (Millipore, 1/200, OP80), mouse anti-CNPase (Millipore, 1/500, MAB326), mouse anti-O4 (R&D systems, 1/100, MAB1326) and rat anti-MBP (Serotec, 1/200, aa82-87) were used to identify more mature oligodendrocytes. Rabbit or mouse anti-GFAP (Dako, 1/500, Z0334 and Sigma, 1/500, G3893) and mouse anti-TUBULIN βIII (Sigma, 1/500, T8578) were used to mark astrocytes and neurons respectively. To assess expression of NESTIN and GLAST in OPCs a monoclonal anti-NESTIN (1/200, Abcam, ab6142) and a guinea pig anti-GLAST (1/200, Millipore, AB1783) antibody were used. Finally, neuroblasts were detected using a rabbit anti-DCX (Abcam, 1/500, ab18723) antibody and SOX2 using the goat anti-SOX2 antibody (Santa Cruz, 1/200, sc-17320). Proliferating cells were identified using a mouse anti-PCNA (Abcam, 1/500, Ab29) antibody and mature olfactory bulb interneurons using rabbit anti-TH (Abcam, 1/500, ab112) and mouse anti-CALRETININ (Abcam, 1/200, ab204990) antibodies. Incubation with blocking buffer (3%BSA, 0.1% Triton x-100 from Sigma, UK, in PBS) and, depending on the requirement, antigen retrieval (15min boiling in 10mM citrate buffer, pH=6.0) preceded incubation with the primary antibodies. The appropriate secondary antibodies were

used, purchased from Invitrogen (Molecular probes, Alexa conjugated 488, 568 and 647), or from Jackson Laboratories (donkey anti-chicken 488 and biotinylated secondary antibodies, as well as avidin-conjugated blue marine fluorophore).

Neurosphere cultures

To grow adult neural stem and progenitor cells *in vitro,* the SEZs of 3 months old tamoxifen-administered mice were dissected, dissociated with papain and plated in high glucose DMEM medium (Gibco), supplemented with B27 (Gibco), N2 (Invitrogen), FGF-2 (20ng/ml, Preprotech), heparin and EGF (20ng/ml, Miltenyi Biotec). Cells were passaged every 7 days and for the differentiation assays 4th passage neurospheres were dissociated and were plated on PDL-coated coverslips, in 24-well plates, in the same medium without growth factors. Cells were fixed (2% PFA, for 20min at room temperature) at 7 and 14 days after plating and were processed for immunocytochemistry as described previously.

Measurements and statistical analysis

For the investigation of the homeostatic CC and SEZ images were acquired with the x40 objective lens and higher magnification details were examined using digital zoom. In each section 5 optical fields were imaged: in the dorsal and middle SEZ, in the dorsolateral tip of the SEZ, in the CC at the middle of the roof of the lateral ventricle and in the CC at the medial tip of the lateral ventricle (in the latter fields the ependymal cell layer was included). The area of the tissue analysed was measured using the Image J software, cell counts were performed using the counter plugin and co-expression was assessed using the Bio-format importer plugin. Co-expression of various molecules was assessed by marking positive cells independently for each immunostaining and subsequently stacking all markings together. For the SEZ, cell numbers were normalized per length of the ventricular wall. In the areas of lesion, at least two optical fields were taken per section, typically from the dorsoventral tip of the SEZ and the CC at the middle of the roof of the lateral ventricle. Only the part of the field containing the lesion, as judged by the presence of high density of cells, was analysed. In the 2 months post-lesion time-point the lesion was usually not visible, therefore measurements were performed according to the co-ordinates of injection, or to the presence of the trait of the needle in the cerebral cortex. Statistical analyses were performed using the Microsoft Office Excel or the SPSS software. When comparing different post-lesion

time-points the one-way ANOVA was used, followed by post-hoc tests. When comparing different post-lesion time-points and ages, the two-way ANOVA was used, followed by post-hoc tests. When comparing sezOPCs and pOPCs for expression of markers, or for densities the student's t-test analysis was used. Statistical significance was always set to $p=0.05$.

EdU toxicity assessment

EdU was injected intraperitoneally twice per day for two days (50µg/g of body weight; 50mg/ml) and mice were culled 4 days after the second injection. EdU was detected following the protocol provided with the Click-iT EdU imaging kits (Invitrogen, Molecular probes) combined with immunohistochemical detection of other markers. In order to compare the possible effects of EdU in the numbers of proliferating cells and of OLIG2+ cells we counted numbers along the whole dorsoventral length of the SEZ using Volocity. In more detail: image stacks were acquired with the Leica TCS SP5 confocal microscope at a z-step spacing of 0.8µm. The software Volocity 6.3.0 (PerkinElmer Inc) was used for counting the number of cells in the distinct channels. For each cell population a minimum object size of $35\mu m^3$ was set and the "separate touching objects" mode was applied. Double positive cells (PCNA+SOX2+, OLIG2+PCNA) were identified as 3D objects of the respective channels intersecting each other, with the resulting population filtered as having a volume above $35\mu m^3$. In both cases the number of cells was verified by manual counting where possible. Results are shown in Table S2 and revealed no significant differences between control and EdU-receiving mice.
