# Supplementary figures and images for: Subependymal Zone-Derived Oligodendroblasts Respond to Focal Demyelination but Fail to Generate Myelin in Young and Aged Mice
Source: Stem Cell Reports. 2017 Feb 9;8(3):685–700. doi: 10.1016/j.stemcr.2017.01.007 (PMC5355571; doi:10.1016/j.stemcr.2017.01.007)

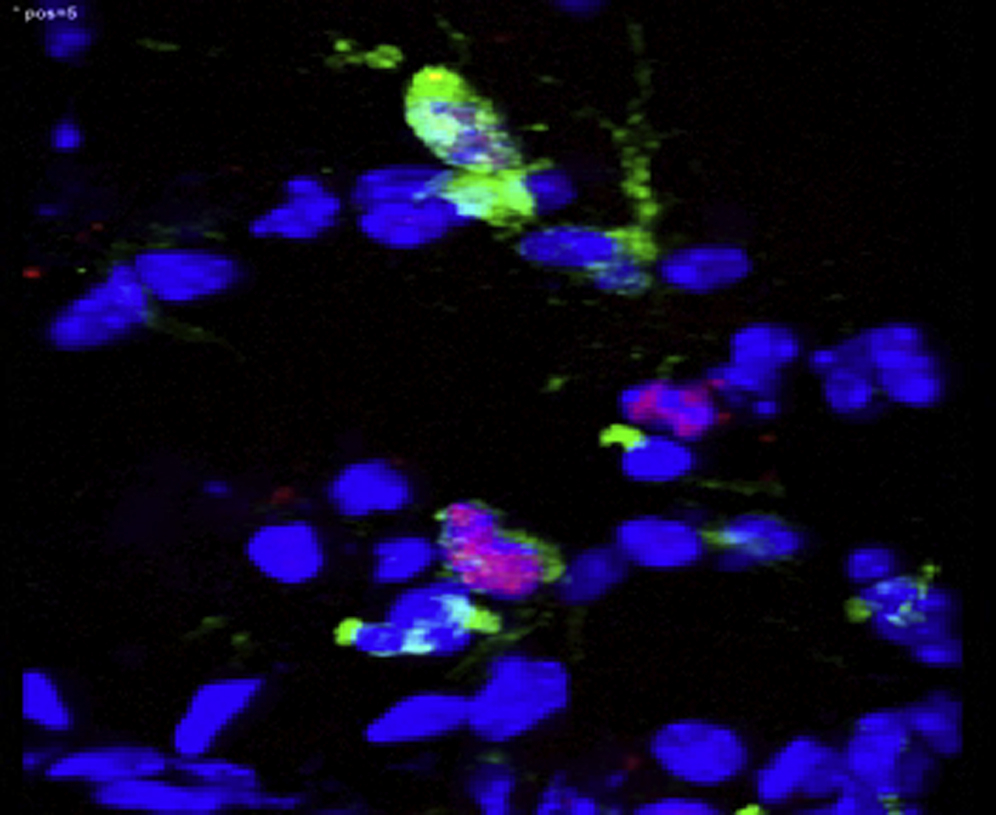

Supplement: Movie S1. 3D Reconstruction of SEZ-Derived OLIG+ Cells [file mmc2.jpg]
